# Effects of the 5:2 intermittent fasting diet on non-alcoholic fatty liver disease: A randomized controlled trial

**DOI:** 10.3389/fnut.2022.948655

**Published:** 2022-07-26

**Authors:** Hamed Kord Varkaneh, Ammar Salehi sahlabadi, Mihnea-Alexandru Găman, Mohsen Rajabnia, Melahat Sedanur Macit-Çelebi, Heitor O. Santos, Azita Hekmatdoost

**Affiliations:** ^1^Department of Clinical Nutrition and Dietetics, Faculty of Nutrition and Food Technology, Shahid Beheshti University of Medical Sciences, Tehran, Iran; ^2^Faculty of Medicine, “Carol Davila” University of Medicine and Pharmacy, Bucharest, Romania; ^3^Center of Hematology and Bone Marrow Transplantation, Fundeni Clinical Institute, Bucharest, Romania; ^4^Gastroenterology and Liver Diseases Research Center, Research Institute for Gastroenterology and Liver Diseases, Shahid Beheshti University of Medical Science, Tehran, Iran; ^5^Department of Nutrition and Dietetics, Faculty of Health Sciences, Ondokuz Mayis University, Samsun, Turkey; ^6^School of Medicine, Federal University of Uberlandia (UFU), Uberlândia, Brazil

**Keywords:** non-alcoholic fatty liver disease, intermittent fasting (5:2), liver enzymes, time-restricted eating, fasting

## Abstract

**Background and aims:**

Dietary regimens are crucial in the management of non-alcoholic fatty liver disease (NAFLD). The effects of intermittent fasting (IF) have gained attention in this regard, but further research is warranted. Thus, we aimed to ascertain the overall effects of the 5:2 IF diet (5 days a week of normal food intake and 2 consecutive fasting days) in patients with NAFLD compared to a control group (usual diet).

**Methods and results:**

A 12-week randomized controlled trial was performed to evaluate the effects of the 5:2 IF diet on anthropometric indices, body composition, liver indices, serum lipids, glucose metabolism, and inflammatory markers in patients with NAFLD. The IF group (*n* = 21) decreased body weight (86.65 ± 12.57–82.94 ± 11.60 kg), body mass index (30.42 ± 2.27–29.13 ± 1.95 kg/m^2^), waist circumference (103.52 ± 6.42–100.52 ± 5.64 cm), fat mass (26.64 ± 5.43–23.85 ± 5.85 kg), fibrosis (6.97 ± 1.94–5.58 ± 1.07 kPa), steatosis scores/CAP (313.09 ± 25.45–289.95 ± 22.36 dB/m), alanine aminotransferase (41.42 ± 20.98–28.38 ± 15.21 U/L), aspartate aminotransferase (34.19 ± 10.88–25.95 ± 7.26 U/L), triglycerides (171.23 ± 39.88–128.04 ± 34.88 mg/dl), high-sensitivity C-reactive protein (2.95 ± 0.62 −2.40 ± 0.64 mg/L), and cytokeratin-18 (1.32 ± 0.06–1.19 ± 0.05 ng/ml) values compared to the baseline and the end of the control group (*n* = 23)—*p* ≤ 0.05 were considered as significant. However, the intervention did not change the levels of high-density lipoprotein cholesterol, total cholesterol, low-density lipoprotein cholesterol, fasting blood sugar, insulin, HOMA-IR, and total antioxidant capacity.

**Conclusion:**

Adhering to the 5:2 IF diet can reduce weight loss and related parameters (fat mass and anthropometric indicators of obesity), as well as hepatic steatosis, liver enzymes, triglycerides, and inflammatory biomarkers in patients with NAFLD.

## Introduction

Non-alcoholic fatty liver disease (NAFLD) has emerged as one of the most prevalent liver disorders worldwide and is believed to affect nearly a quarter of the global population, translating into an increased economic burden on healthcare systems (1). Although genetic factors also play a pivotal role in the development of NAFLD, a major contribution to its pathogenesis is attributed to modifiable risk factors, i.e., obesity, metabolic syndrome, and diabetes (2, 3). As patients diagnosed with NAFLD are prone to developing liver cirrhosis as well as hepatocellular carcinoma, urgent measures are needed to combat the spread of the NAFLD epidemic (4–7), particularly as there are no approved pharmacotherapies for this chronic hepatic disorder.

Dietary interventions remain an attractive strategy in the management of NAFLD and cardiometabolic problems, especially due to the improvement in indicators of adiposity, serum lipids, glycemic control, and blood pressure (8–13). In recent years, IF regimens have gained massive popularity in the field of clinical nutrition as a method of energy restriction, possibly because patients are more likely to accept and adhere to lifestyle changes than taking pills daily (14–17). The 5:2 diet is an emerging type of periodic fasting in which individuals fast 2 days a week and eat freely for 5 days a week (18, 19). Time-restricted feeding and the 5:2 diet are all close to mandatory and voluntary Islamic fasting, with time differences (20, 21). More specifically, regarding the adherence to the 5:2 diet, there is substantial energy restriction on the fasting days, in which it is common to prescribe 0–25% of the estimated total daily energy requirements on 2 non-consecutive days of the week without water restriction, while the habitual intake (*ad libitum*) or 100% of estimated total daily energy requirements are used for the remaining 5 days (19).

Randomized controlled trials (RCTs) have examined the effects of the 5:2 diet in different populations. Fudla et al. reported that participants with obesity on the 5:2 diet for 1 month experienced a reduction in body mass index (BMI) (22). Increasing attention has been paid to the effects of IF regimens on the management of NAFLD, as shown by a systematic review and meta-analysis of RCTs supporting reductions in body weight, BMI, as well as liver enzymes in individuals on IF diets (Ramadan fasting, alternate-day fasting, and time-restricted feeding) for 4–12 weeks (23). More recently, Holmer et al. (7) found that adherence to the 5:2 diet improves indicators of obesity, lipid and glycemic indices, and hepatic steatosis in patients with NAFLD (7). However, Holmer et al. did not assess body composition and inflammatory biomarkers, and did not calculate the basal metabolic rate individually; instead, they used general energy intake recommendations based on sex (7).

Thus, we conducted a 12-week RCT to examine the effects of the 5:2 diet on patients with NAFLD, employing assessment of traditional parameters (imaging and blood tests) of this disease, cardiometabolic and inflammatory biomarkers, body composition, and anthropometric indicators of obesity. Our hypothesis was that the 5:2 diet could significantly improve some cardiometabolic and hepatic markers due to the accompanying weight loss.

## Methods and materials

We recruited patients diagnosed with NAFLD and overweight/obesity between October 2019 and May 2020 from the Gastroenterology and Liver Diseases Research Center, Tehran, Iran. The following inclusion criteria were applied: BMI = 25–40 kg/m^2^, age between 18 and 50 years (as middle-aged and elderly participants are less available for fasting), grade 2 NAFLD, —i.e., a controlled attenuation parameter (CAP) score ≥ 260 dB/m and ≥ 34% fatty change, with liver stiffness measurement (LSM) levels <14 kPa (24)—, willingness to participate in the RCT, and no recent participation in a weight loss diet program.

Based on the BMI range, there was exclusion of participants at underweight and healthy levels, as well as Class 3 obesity, to provide greater uniformity. For instance, the patients at Class 3 obesity generally require more calorie in a traditional dietary model, and, thus, enrollment in an IF regimen is difficult in virtue of the shorter feeding window. Regarding the age range, we aimed to avoid middle-aged and elderly participants due to the lower availability of this population for fasting or adherence to new dietary models that yield habitual changes. In addition, the patients with NAFLD Grade 3 were not included because IF regimens putatively might be dangerous for this population due to the pathophysiological link between the very high liver accumulation of triglycerides (TG) and its worsening with rapid mobilization and oxidation of TG in this organ during fasting (25, 26).

Regarding medication use, the patients should not have used antibiotics for more than 1 week during the study period or prior to enrollment, and should not have consumed herbal medicines, anti-inflammatory drugs, corticosteroids/any type of hormone, weight-loss drugs, or hepatotoxic drugs, e.g., phenytoin, amoxicillin, and lithium. In addition, the patients should not be alcohol consumers or diagnosed with cardiovascular diseases, stroke, diabetes, acute liver disorders (hepatitis B, C, etc.), kidney diseases, chronic inflammatory disorders, depression, cancer, or autoimmune diseases. The RCT only included the participants who met the aforementioned criteria and who were willing to adhere to a 12-week program of the 5:2 IF diet.

We applied the following exclusion criteria: weight loss ≥ 10% or weight gain ≥ 5% in the last 6 months, use of pharmacological agents that can affect metabolism or liver function (e.g., lipid-lowering or glucose-lowering drugs, drugs used to control blood pressure) or supplements/substances that may interact with the impact of diet during the intervention.

Before enrollment in the RCT, all the patients had signed informed consent form. We registered the RCT at www.irct.ir (IRCT20100524004010N31). This study was approved by the Ethics Committee of the Faculty of Nutrition and Food Technology, Shahid Beheshti University of Medical Sciences, Tehran, Iran (IR.SBMU.NNFTRI.REC.1399.019).

## Study design and randomization

### Participant recruitment

The study was designed as an RCT, with blinding for randomization at the study clinic. Eligible individuals suffering from NAFLD were stratified based on age and BMI and then randomized using a computer-generated random-numbers method to the IF (5:2) group (*n* = 24) or a non-interventional control group (*n* = 25).

### Sample size

The number of samples required for this study was calculated based on different dependent variables. The highest number of samples was obtained for the cap score-dependent variable. The sample size for this study was based on how many samples should be selected so that the average cap score difference between the 5:2 diet and the control group is at least 25 units per liter. This difference when reaching a probability of 95% (α = 0.05) and power of 80% (β = 20%) was considered statistically significant. The number of samples for each group was estimated to be 21 patients. The sample size was calculated based on the deviation from the criteria obtained in a previous study [86] and using the following formulas.


$$\begin{array}{lll} n_{1} & = & 8S_{1}=\;34\\ n_{2} & = & 8S_{2}=\;23\\ \mu_{1} & = & \mu_{2}=\;25\\ Z_{1-\alpha /2} & = & =\;1/96\\ Z_{1-\beta } & = & 0/84\\ S_{\text{p}}^{2} & = & \frac{\left(n_{1}-1\right)S_{1}^{2}+\;\left(n_{2}-1\right)S_{2}^{2}}{n_{1}+n_{2}-\;2}=\;842/5\\ n & = & \frac{2S^{2}{\left(Z_{1-\alpha /2}+Z_{1-\beta }\right)}^{2}}{{\left(\mu_{1}-\mu_{2}\right)}^{2}}=\;21 \end{array}$$

### Dietary interventions

The intervention group was allocated to adhere to the 5:2 diet, i.e., 5 days a week of normal food intake and 2 consecutive fasting days (Monday and Tuesday) a week for 12 weeks. On fasting days, the patients received 25% of the recommended calorie intake over a 2-h period from 12:00 to 2:00 p.m. Such a calorie goal was divided into 30% calories from fat, 15% calories from proteins, and 55% calories from carbohydrates.

The 5:2 diet was selected as it seemed more convenient when compared to other IF regimens, e.g., alternate-day or Ramadan-style fasting. The patients allocated to the control group were asked to follow to their usual diet. Data were evaluated by a registered dietitian before the start of the RCT, whose professionals were responsible for body composition, questionnaires, and dietary prescription.

The caloric intake of the patients was evaluated using the Mifflin-St Jeor Equation (27). For both groups, the intake of macronutrients was: 30% calories from fat, 15% calories from proteins, and 55% calories from carbohydrates. Consumption of non-caloric fluids, e.g., water, coffee, or tea, was allowed for the intervention and control groups, and the patients were advised to drink water without restriction during the RCT. Thus, these non-caloric fluids were allowed for fasting and feeding periods.

A diet plan for 3 months was prescribed for each subject. At the beginning of the RCT, all the patients were instructed on how to adhere to the diet and were monitored during the study by weekly phone calls and monthly interviews based on three 24-h recalls. The patients who did not adhere to the diet were dropped from the RCT.

### Measurements

All the patients had the body weight, height, waist circumference (WC), and body composition measured at the beginning and the end of the RCT. Fasting blood samples were collected in the morning after 10–12 h of fasting. Liver steatosis and fibrosis were evaluated using the FibroScan® 502 Touch device at the beginning of the RCT end of 12 weeks. In addition, physical exercise questionnaires (28), 24-h recalls (29, 30), and questionnaires related to patients' demography data were completed. Nutritionist IV software was employed to assess the dietary energy and macronutrient content.

### Anthropometric measurements and body composition

We evaluated body weight using a digital scale (Seca 808, Germany; ±0.1 kg accuracy), with patients wearing light clothing and barefooted. WC was assessed at the midpoint between the 12th rib and the iliac crest and during exhalation. The standing height of the patients was evaluated to the nearest 0.5 cm, without shoes, using a wall stadiometer (Seca, Germany) and by standard procedures.

We assessed fat mass, lean body mass (LBM), and total body water (TBW) using bioelectrical impedance analysis (BIA) (Tanita-BC 418 MA, Arlington Heights, USA) at the beginning and the end of the RCT. The evaluation was performed after 12 h of fasting. The patients were asked not to perform physical exercise 12 h before the test to empty the urinary bladder 30 min before the measurements and to remove metallic objects immediately before the test. The assessment was performed outside the menstrual period for women of childbearing age as well.

### Blood sample measurements

We collected blood samples (10 ml) from the participants between 7 and 10 A.M., then the probes were centrifuged at 2,000 g (RCF) at room temperature for 20 min. Serum biomarkers were assessed at the beginning and the end of the RCT.

Liver enzymes [alanine transaminase (ALT), aspartate transaminase (AST), and gamma-glutamyl transferase (GGT)] and the lipid profile [total cholesterol (TC), high-density lipoprotein cholesterol (HDL-C), LDL-C, and TG] were measured following standard procedures recommended by Delta-dp diagnostic kits (Roche, Germany).

Inflammatory biomarkers [high-sensibility C-reactive protein (hs-CRP) and cytokeratin-18 (CK-18)] and total antioxidant capacity were evaluated using the ELISA method (ZellBio GmbH, Ulm, Germany).

FBS concentrations were assessed using an auto-analyzer by the glucose oxidase method (Cobas c311, Roche Diagnostics, Risch-Rotkreuz, Switzerland). Serum insulin levels were assessed using ELISA kits (Monobind Inc., Lake Forest, California, USA). In order to assess insulin resistance, we calculated the homeostasis model assessment of insulin resistance (HOMA-IR) by using the following formula: fasting insulin (μU/L) x fasting glucose (nmol/L)/22.5.

### Statistical analysis

SPSS software version 22 was employed to compute the statistical analysis. Data were reported as mean ± standardized deviation (SD). The Kolmogorov-Smirnov test was employed to evaluate dara compliance with the normal distribution. We only evaluated data from the patients who completed the RCT (i.e., per-protocol analysis).

One-way ANOVA and independent samples *t*-tests were used to compare quantitative variables between groups at the baseline. The chi-squared test was employed to evaluate qualitative variables. An independent sample *t*-test was employed to compare the quantitative variables between groups before and after the intervention.

The Student's *T*-test/paired samples *t*-test was employed to compare quantitative variables within each of the two groups before and after the intervention. Analysis of covariance (ANCOVA) was performed to compare the mean of variables between groups by adjusting for confounding factors (BMI, body weight; WC, metabolic equivalents; dietary energy intake; and baseline value of the outcomes). The statistical significance was set at *p* ≤ 0.05.

## Results

**Figure 1:**
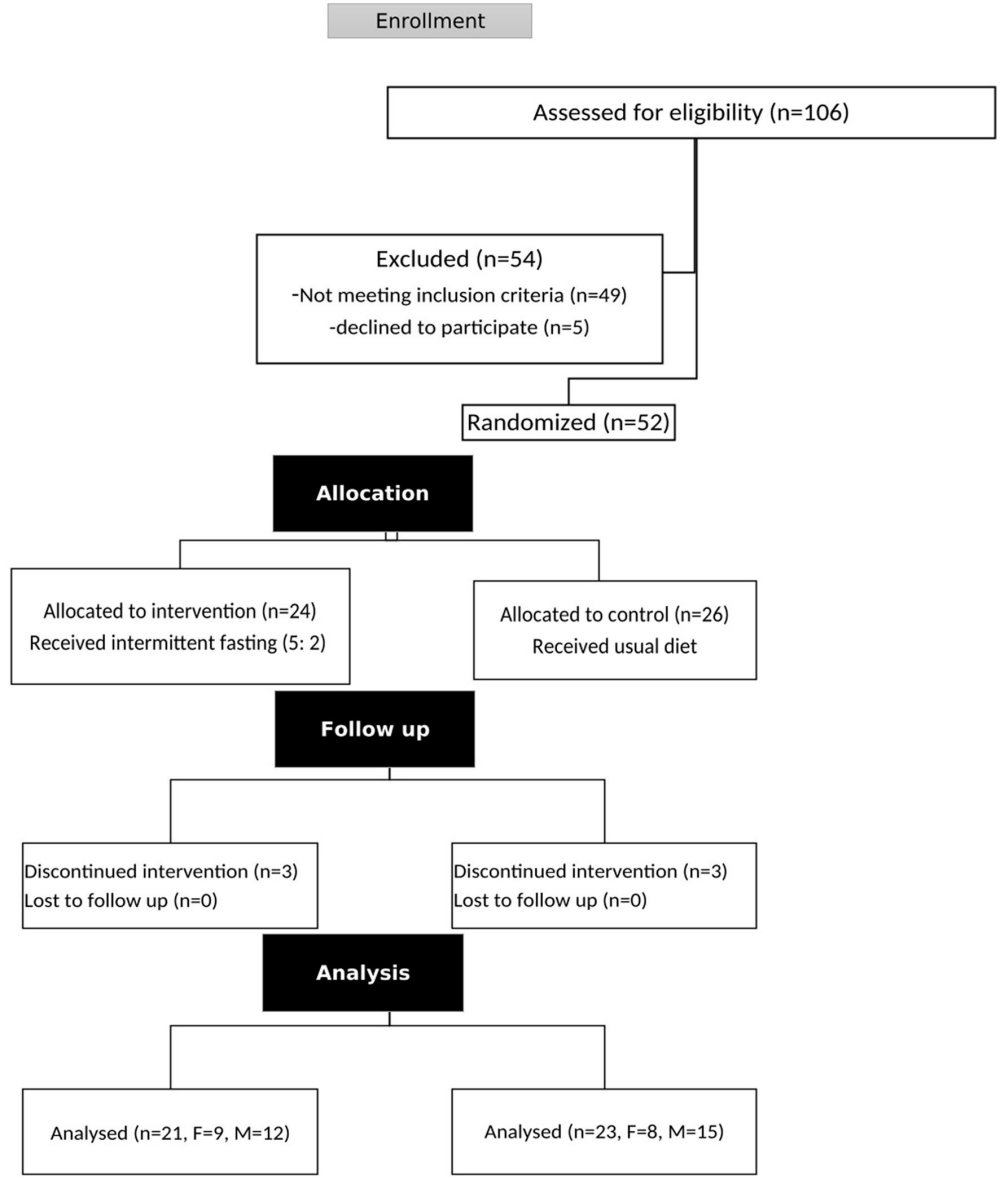
Flowchart of the participants evaluated.

Three patients dropped out of the intervention group and three patients dropped out of the control group during the 12-week RCT. A total of 21 and 23 individuals completed the intervention and control group procedures, respectively (Figure 1). The mean age, BMI, WC, and body weight of the patients were 45.25 ± 9.84 years, 30.51 ± 2.7 kg/m^2^, 105.59 ± 8.11 cm, and 88.05 ± 11.88 kg, respectively. The baseline characteristics of the patients were similar between the intervention and control groups (Table 1).

**Table 1:** Baseline characteristics, and anthropometric indices of the participants.

**Characteristics**	**Intermittent fasting (5:2) diet (*****n*** = **21)**	**Control diet (*****n*** = **23)**	***P*** **-value** ^b^
Age (years)	46.42 ± 13.35	44.17 ± 4.9	0.454
**Gender n (%)**			
Male	12 (57.1)	15 (65.2)	0.405
Female	9 (42.9)	8 (34.8)	
**Smoking n (%)**			
Yes	2(9.5)	2(8.7)	0.661
No	19(90.5)	21(91.3)	
**Anthropometric indices**			
Height (cm)	168.47 ± 10.68	170.91 ± 9.83	0.435
Body weight (kg)
Before	86.65 ± 12.57	89.33 ± 18.47–88.31 ± 11.01	0.461
After	82.94 ± 11.60	88.31 ± 11.01	0.123
*P*-value^a^	<0.001	0.064	
BMI (kg/m^**2**^)
Before	30.42 ± 2.27	30.60 ± 30.09	0.826
After	29.13 ± 1.95	30.26 ± 3.08	0.158
*P*-value^a^	<0.001	0.063	
Waist circumference (cm)
Before	103.52 ± 6.42	107.09 ± 9.47	0.107
After	100.52 ± 5.64	107.19 ± 8.93	0.006
*P*-value^a^	0.001	0.928	
Lean body mass (kg)
Before	59.97 ± 10.75	62.10 ± 11.29	0.525
After	59.09 ± 10.37	59.33 ± 12.28	0.854
*P*-value^a^	0.565	<0.0001	
Body fat (kg)
Before	26.64 ± 5.43	27.21 ± 7.33	0.762
After	23.85 ± 5.85	28.93 ± 7.91	0.025
*P*-value^a^	0.039	0.041	
Total body water (kg)
Before	43.76 ± 8.21	45.11 ± 8.57	0.140
After	43.11 ± 7.37	43.19 ± 7.87	0.136
*P*-value^a^	0.773	0.001	

*BMI, body mass index*.

*Values are expressed as mean ± SD*.

a *Within-group comparison (a paired samples t-test was used for testing two values within group)*.

b *Between-group comparison (an independent sample t-test was used for quantitative variables)*.

### Anthropometric indices and body composition

Data regarding the anthropometric indices and body composition of the patients are depicted in Table 1. Following the intervention, the individuals who underwent a 5:2 diet experienced a significant decrease in body weight (−3.7 kg on average; 86.65 ± 12.57 kg to 82.94 ± 11.60 kg, *p* < 0.001), while there was a non-significant reduction in the control group (−1.02 kg on average, 89.33 ± 18.47 to 88.31 ± 11.01 kg, *p* = 0.06). Moreover, the individuals on the 5:2 diet experienced a significant decrease in BMI (30.42 ± 2.27–29.13 ± 1.95 kg/m^2^), WC (103.52 ± 6.42–100.52 ± 5.64 cm), and fat mass (59.97 ± 10.75–59.09 ± 10.37 kg) vs. the control group.

### Nutritional intake and physical activity

Information on dietary intake and physical activity (MET.hr/day) of participants is depicted in Table 2, where the dietary data at the end for the intervention group were an average of 5 days of feeding and 2 days of fasting. The intervention and control groups had similar physical activity levels at the end of intervention vs. the baseline (the intervention group: 29.48 ± 5.60 vs. 29.99 ± 5.15 MET.hr/day, *p* > 0.05; the control group: 30.38 ± 5.13 vs. 30.52 ± 5.19 MET.hr/day, *p* > 0.05). Inter-group differences were not detected. Both intervention and control groups had a significant decrease in total energy intake vs. the baseline (the intervention group: 2,862.95 ± 721.60–2,601.57 ± 726.55 kcal/day, *p* < 0.001; the control group: 3,120.91 ± 733.27–2,967.30 ± 665.80 kcal/day, *p* = 0.001). No significant inter-group differences were detected. The intervention group decreased total protein intake vs. the baseline (93.38 ± 35.32–84.67 ± 25.74 g/day, *p* < 0.001) but did not change for other dietary variables.

**Table 2:** Dietary intakes and physical activity.

**Variables**	**Baseline**	**End of the study** *	***P*** **-value** ^a^
**Physical activity (MET hr/day)**			
Intervention	29.48 ± 5.60	29.99 ± 5.15	0.189
Control	30.38 ± 5.13	30.52 ± 5.19	0.245
*P*-value^b^	0.531	0.801	
**Total energy (kcal/day)**			
Intervention	2,862.95 ± 721.60	2,601.57 ± 726.55	<0.001
Control	3,120.91 ± 733.27	2,967.30 ± 665.80	0.001
*P*-value^b^	0.247	0.089	
**Total carbohydrate (g/day)**			
Intervention	459.00 ± 127.81	433.29 ± 106.48	0.096
Control	446.17 ± 109.71	437.00 ± 115.66	0.597
*P*-value^b^	0.722	0.913	
**Total protein (g/day)**			
Intervention	93.38 ± 35.32	84.67 ± 25.74	0.022
Control	112.96 ± 28.47	107.57 ± 23.34	0.164
*P*-value^b^	0.048	0.003	
**Total fat (g/day)**			
Intervention	104.90 ± 34.55	102.67 ± 37.20	0.634
Control	116.39 ± 35.36	111.83 ± 28.07	0.408
*P*-value^b^	0.283	0.359	
**Dietary fiber (g/day)**			
Intervention	48.05 ± 11.26	43.38 ± 9.64	0.170
Control	47.96 ± 14.54	48.83 ± 13.07	0.742
*P*-value^b^	0.982	0.126	

*MET, metabolic equivalents*.

a *Within-group comparison (a paired samples t-test was used for testing two values within group)*.

b *Between-group comparison (an independent sample t-test was used for quantitative variables)*.

* *Dietary data at the end for the intervention group were an average of 5 days of feeding and 2 days of fasting*.

### Liver parameters

The results of the assessment of liver parameters are reported in Table 3. Fibrosis and steatosis scores decreased after the 5:2 diet (6.97 ± 1.94–5.58 ± 1.07 kPa and 313.09 ± 25.45–289.95 ± 22.36 dB/m, respectively) and reached lower levels than the control group (*p* = 0.040 and *p* = 0.042, respectively). In addition, the patients on the 5:2 diet had a significant reduction in ALT (41.42 ± 20.98–28.38 ± 15.21 U/L, *p* = 0.043) and AST (34.19 ± 10.88–25.95 ± 7.26 U/L, *p* = 0.01) levels vs. the control group (*p* < 0.05).

**Table 3:** Lipid profile, glycemic indices, liver enzymes, inflammatory biomarkers, and indices of hepatic steatosis and fibrosis.

**5:2 Intermittent fasting diet**			**Control diet**				***P*** **-value** ^b^
**Variables**	**Baseline**	**End**	***P*** **-value** ^a^	**Baseline**	**End**	***P*** **-value** ^a^	
**Liver parameters**							
ALT (U/L)	41.42 ± 20.98	28.38 ± 15.21	0.043	30.34 ± 5.13	28.04 ± 8.12	0.144	0.044
AST (U/L)	34.19 ± 10.88	25.95 ± 7.26	0.013	23.39 ± 8.13	23.77 ± 9.66	0.969	0.015
GGT (U/L)	31.09 ± 26.25	19.52 ± 7.15	0.053	34.77 ± 12.93	39.22 ± 36.95	0.532	0.080
Fibrosis score (kPa)	6.97 ± 1.94	5.58 ± 1.07	0.009	5.82 ± 1.44	5.46 ± 1.32	0.072	0.040
Steatosis score/CAP dB/m	313.09 ± 25.45	289.95 ± 22.36	<0.001	311.52 ± 33.65	306.00 ± 37.35	0.342	0.042
**Lipid profile**							
TG (mg/dL)	171.23 ± 39.88	128.04 ± 34.88	<0.001	187.6 ± 73.61	199.56 ± 87.43	0.512	<0.001
TC (mg/dL)	165.38 ± 26.06	164.00 ± 22.83	0.865	172.21 ± 37.99	180.72 ± 49.49	0.425	0.285
HDL-C (mg/dL)	41.22 ± 11.64	38.68 ± 11.13	0.079	34.82 ± 7.65	33.73 ± 6.70	0.290	0.167
LDL-C (mg/dL)	90.11 ± 26.50	93.07 ± 28.91	0.551	93.73 ± 31.77	97.45 ± 35.46	0.734	0.015
**Glycemic indices**							
FBS (mg/dL)	106.31 ± 28.11	101.11 ± 20.46	0.321	102.82 ± 11.71	105.78 ± 14.16	0.230	0.523
Insulin (mU/L)	10.95 ± 3.24	12.18 ± 4.26	0.058	10.78 ± 3.78	12.15 ± 5.25	0.219	0.898
HOMA-IR	3.02 ± 1.34	2.95 ± 1.35	0.528	2.77 ± 1.11	3.15 ± 1.41	0.269	0.249
**Inflammatory and antioxidant biomarkers**							
hs-CRP (mg/L)	2.95 ± 0.62	2.40 ± 0.64	<0.001	2.72 ± 1.04	2.75 ± 1.13	0.716	<0.001
CK-18 (ng/mL)	13.23 ± 0.61	11.92 ± 0.53	<0.001	13.24 ± 2.76	18.56 ± 3.54	<0.001	<0.001
TAC (mmol/L)	0.35 ± 0.06	0.33 ± 0.09	0.341	0.30 ± 0.8	0.32 ± 0.0.09	0.451	0.296

*ALT, alanine aminotransferase; AST, aspartate aminotransferase; CAP, controlled attenuation parameter; CK-18, cytokeratin-18; FBS, fasting blood sugar; GGT, γ-glutamyltransferase; HDL-C, high-density lipoprotein cholesterol; HOMA-IR, homeostasis model assessment of insulin resistance; hs-CRP, high-sensitive C-reactive protein; LDL-C, low-density lipoprotein cholesterol; TAC, total antioxidant capacity; TC, total cholesterol; TG, triglycerides*.

a *Within-group comparison (a paired samples t-test was used for testing two values within group)*.

b *Analysis based on ANCOVA model for assessing the regression relationship of variables between the groups at the end of the study after adjustments for body mass index, weight, waist circumference, metabolic equivalents, dietary energy intake, and baseline value of the outcome*.

### Lipid profile

TG concentrations (171.23 ± 39.88 mg/dL to 128.04 ± 34.88 mg/dL, *p* < 0.001), but not HDL-C, TC, or LDL-C, decreased significantly in the intervention vs. the control group (*p* < 0.001) (Table 3).

### Glycemic indices

The decrease in FBS values in the intervention group (106.31 ± 28.11–101.11 ± 20.46 mg/dL) was not statistically significant vs. the control group (*p* = 0.52). Moreover, insulin levels and HOMA-IR values did not change significantly in the intervention or control group (Table 3).

### Inflammatory biomarkers

After the intervention, the participants on the 5:2 diet exhibited a decrease in hs-CRP (2.95 ± 0.62–2.40 ±0.64 mg/L, *p* < 0.001) and CK-18 (13.23 ± 0.61–11.92 ±0.53 ng/ml, *p* < 0.001) levels vs. the control group (Table 3). On the other hand, the total antioxidant capacity did not change significantly following the RCT.

## Discussion

This RCT shows that the 5:2 diet can be a non-pharmacological tool in the management of NAFLD by improving not only specific markers of NAFLD but also several cardiometabolic parameters. More specifically, we demonstrated significant reductions in body weight, BMI, WC, body fat, ALT, AST, fibrosis and steatosis scores, TG, hs-CRP (mg/L), and CK-18 (ng/ml) after 12 weeks on the 5:2 diet. On the other hand, the 5:2 diet did not alter the levels of HDL-C, TC, LDL-C, glycemic markers (FBS, insulin, and HOMA-IR), and total antioxidant capacity. Taken together, these results are in line with our hypothesis, given that we expected some improvements in cardiometabolic and hepatic markers with accompanying weight loss and decreased adiposity. So much so that massive research shows beneficial effects of different IF regimens in this regard (31–37).

Different types of IF regimens have been used to improve body composition and anthropometric indices in patients with NAFLD, from the Ramadan fasting to the 5:2 diet. Ramadan is a type of IF regimen that can reduce body weight, body fat, BMI, and WC in patients with NAFLD compared to non-fasting (38), but, overall, our results tend to be better due to the longer length of intervention since Ramadan lasts approximately 4 weeks. Our findings are more similar to the study by Holmer et al. (7), who performed an RCT for patients with NAFLD (*n* = 74) and observed a decrease in body weight by 7.4 kg and in BMI by 2.4 kg/m^2^ over a 12-week period on a 5:2 diet, whose effect was similar to a low-carbohydrate high-fat diet and better than a standard of care (7). We observed a lower decrease in body weight (−3.7 kg) and BMI (−1.3 kg/m^2^) lower than in the study by Holmer et al. This was probably because there was a mean reduction of 590 kcal/d at the end of the study by Holmer et al., whereas we observed a mean reduction of 260 kcal. It must be noted that Holmer et al. prescribed the 5:2 diet, guiding a daily intake of 500 kcal for 2 non-consecutive days a week, followed by a general recommendation based on an intake limit of 2,000 kcal/d for women and 2,400 kcal/d for men, while we estimated the patients' basal metabolic rate using the Mifflin-St Jeor Equation and, therefore, providing more reliable data in terms of individual care. Moreover, we observed reductions in WC (−3 cm) and fat mass (−2.8 kg), while, seemingly, Holmer et al. did not assess these parameters.

It is important to note that both the intervention and control groups had a significant decrease in total energy intake compared to the baseline (the intervention group: ~2,863– ~ 2,602 kcal/day, *p* < 0.001; the control group: ~3,121–2,967 kcal/day, *p* = 0.001), but without the inter group differences (*p* < 0.05). In summary, there was a modest reduction in calorie intake in both groups (261 kcal and 154 kcal for intervention and control groups, respectively). Although the control group was instructed to consume the habitual diet, there was a reduction in calorie intake that probably may have occurred because the participants were part of a clinical study. However, reduced calorie intake on the 5:2 diet had a greater clinical magnitude than the control group regardless of the absence of significant intergroup difference, as the 5:2 diet was more effective in improving body composition and laboratory parameters. It is crucial to highlight that we adjusted the intergroup variables at the end of the study for BMI, body weight, WC, and energy intake.

Reducing energy intake can be considered the main mechanism leading to improvement in adiposity parameters and cardiometabolic markers (38). Decreased energy intake within an IF diet plan may promote mobilization of free fatty acids, increase fat oxidation and production of ketones, as well as can improve circadian rhythm by modulating clock genes and anti-inflammatory molecules with a myriad of metabolic improvements (e.g., melatonin) when the dietary strategy creates a health routine for the subject (39–43). Regarding the molecular basis involving the interplay between liver and lipids, Santos and Macedo suggest that IF reduces the production of apolipoprotein B and thereby increases fatty acid oxidation and decreases hepatic TG content, yielding a reduction in circulating levels of very-low-density lipoprotein cholesterol, LDL-C, and small-dense LDL-C (44). The latter parameter shed light on the anti-atherogenic effects of IF diets, as the small-dense LDL-C represents the LDL particles with highest affinity for arterial damage (45). In contrast, there is a debate about the effects of IF regimens on improving lipid indices, as discussed thoroughly below.

Employing an alternate-day fasting, i.e., alternating between an *ad libitum* feeding day and a 75% energy-restricted fasting day, Cai et al. (46) found improvement in TC and TG within a relatively short length of intervention (4–12 weeks) in patients with NAFLD, but LDL-C and HDL-C levels did not change (46). Holmer et al. found a decrease in TG, TC, and LDL-C levels by 35, 19, and 14 mg/dL, respectively, in patients with NAFLD undergoing a 5:2 diet for 12 weeks, while HDL-C did not alter (7). In our study, we found a decrease in TG (~43 mg/dL), with a clinical magnitude similar to the reduction by Holmer et al., but we did not observe changes in other lipid parameters.

In patients with NAFLD, liver tests (ALT, AST, alkaline phosphatase, and GGT) are routinely included in the clinical evaluation. We demonstrated a decrease in ALT and AST levels by 13 and 8 U/L, respectively, after the diet 5:2. Johari et al. (47), in turn, found a decrease in ALT and AST levels by 25 and 8 U/L, respectively, in patients with NAFLD after 8 weeks of alternate-day calorie restriction (47). The authors explained this reduction in liver enzymes by the improvement of visceral fat or steatosis in the liver (47). Likewise, we demonstrated improvement in steatosis and fibrosis scores alongside a decrease in GGT levels (~11 U/L). Collectively, these results can be considered the central tenet of our research, as the primary goal of NAFLD treatment is to improve specific parameters of this ailment, which is secondarily associated with several metabolic benefits.

Increased levels of inflammatory biomarkers in patients with NAFLD might affect the pathogenesis of cardiovascular diseases. CRP is a protein produced by the liver under stimulation of pro-inflammatory cytokines, and the hs-CRP assay is a recommended marker of low-grade inflammation for screening the risk of cardiovascular diseases (48–50). It is no wonder that hs-CRP is a non-invasive complementary marker of NAFLD, and its high levels are common in this population due to the interplay between fatty liver and cardiometabolic problems (51). Aliasghari et al. (38) reported decreased levels of hs-CRP after Ramadan fasting (38). In our research, the group that underwent a 5:2 diet had lower levels of hs-CRP at the end of the study both compared to the baseline (−0.6 mg/L) and end levels of the control group (−0.4 mg/L). CK-18 is another inflammatory marker that circulates as a consequence of oxidative stress, hepatocyte apoptosis, or inflammation in NAFLD (52). In a meta-analysis, CK-18 levels proved to be an important tool for diagnosing NAFLD, especially non-alcoholic steatohepatitis (52), and, in our work, we showed a reduction in CK-18 levels after the 5:2 diet. The reduction in CK-18 levels after the 5:2 diet is not impressive, but, at least, it has a modest clinical magnitude, as we observed a reduction from ~13.2 to 11.9 ng/ml, and a proposed CK-18 cutoff value to detect steatosis (S ≥ 2) in NAFLD is 11.7 ng/ml (53).

Recently, in 2022, IF has been highlighted as a safe and effective tool to improve liver histology in NAFLD (54). Behind the scenes of histology, fasting-induced AMP-activated protein kinase (AMPK) is the main biological rationale in this regard, whose enzyme is a master regulator of energy metabolism that activates fatty acid oxidation and breakdown (54), but it is crucial to know that this mechanism is enhanced by carbohydrate restriction and mainly by skeletal muscle contraction during exercise (55). Hepatic steatosis and fibrosis are two of the most studied histological parameters due to their importance in disease diagnosis and staging (56). In patients with NAFLD, excess visceral fat lipids and accompanying inflammation sharply increase the risk of developing complications of chronic liver disease, such as cirrhosis, liver failure, and hepatocellular carcinoma (57). Advanced fibrosis is the most significant predictor of mortality in NAFLD (58). In our study, since 5:2 was associated with improved fibrosis and steatosis scores, we provide evidence that such a dietary regimen is a viable non-pharmacological strategy to improve key clinical parameters that are a result of NAFLD pathology, but RCTs performing liver biopsies are needed to analyze the NAFLD histology.

Type 2 diabetes is a recognized risk factor in the NAFLD severity (59). Patients with NAFLD who underwent Ramadan fasting had reduced FBS, insulin, and HOMA-IR levels but not in the non-fasting group (38). Overall, in a meta-analysis consisting of 12 articles (545 participants), IF diets were associated with a significant decline in FBS (weighted mean difference, −4.16 mg/dL; 95% confidence interval, −6.92– −1.40) when compared with a control diet (60). In our study, we did not find significant changes in FBS, insulin, and HOMA-IR. These null effects may be a result of many patients in our sample being normoglycemic, but mean FBS levels were within the range of pre-diabetes, and this fact cannot be ruled out. At best, there was a non-statistical decrease in FBS by ~5 mg/dl after the 5:2 diet that nearly corrected mean FBS levels from the pre-diabetic stage to normoglycemia (from ~106 to ~101 mg/dl).

Current guidelines suggest that dietary intervention and exercise are first-line therapy for NAFLD (61, 62). Correspondingly, the European Association for the Study of the Liver (EASL) guidelines focus on lifestyle changes through diet and habitual physical activity and emphasize that diet-induced weight loss is the only treatment to ameliorate liver damage without severe liver fibrosis (63). Given that these guidelines recommend a pragmatic and individualized approach and do not advocate any specific diet for the treatment of NAFLD, our findings are noteworthy as a means of expanding the effect of IF regimens as one of many dietary strategies that can be employed through a personalized perspective, but this does not imply that IF is the best dietary approach. Bearing in mind the importance of personalized dietary plans, the addition of functional foods and supplements could also enhance the effect of IF or other dietary patterns in the management of liver parameters and cardiometabolic markers (64–70).

As for strengths, our data reach different spheres of medicine, as we observed improvement in several laboratory markers with clinical relevance. For instance, (i) ALT and AST are biomarkers for screening liver function; (ii) fibrosis and steatosis are important determinants of NAFLD diagnosis (71); (iii) associated atherogenic dyslipidemia is often observed with increased plasma levels of TG in patients with NAFLD, and cardiovascular diseases are the most common causes of mortality in this population (72); (iv) hs-CRP is recommended for coronary risk assessment in adults and is significantly higher in patients with NAFLD (51); (v) CK-18 (ng/ml) is one of the biomarkers in the circulation as a result of oxidative stress, hepatocyte apoptosis, or inflammation in response to lipid metabolism disorders because of NAFLD (52). In addition, we assessed body composition alongside anthropometric indicators of obesity that is easily replicated in clinical practice and used in nutrition science (73–76).

As for limitations, we did not use gold-standard methods for body composition and energy expenditure nor did we perform liver biopsies. In addition, we used a physical activity questionnaire to assess physical activity. Ultimately, we encourage further research focusing on NAFLD and accompanying diseases, such as dyslipidemia and diabetes, to better expand the clinical magnitude of the 5:2 diet and other types of IF regimens.

## Conclusion

The 5:2 diet can reduce weight loss and related parameters (fat mass and anthropometric indicators of obesity), as well as hepatic steatosis, liver enzymes, TG, and inflammatory biomarkers (hs-CRP and CK-18) in patients with NAFLD. However, the intervention did not change the levels of HDL-C, TC, LDL-C, glycemic markers, and total antioxidant capacity. In other words, the 5:2 diet can be a non-pharmacological tool in the management of NAFLD by improving not only specific markers of NAFLD but also several cardiometabolic parameters. Nevertheless, the 5:2 diet cannot be considered the best dietary regimen in this regard, but deserves attention as one of the many strategies that integrate the arsenal of nutrition-based approaches.

## Data Availability Statement

The original contributions presented in the study are included in the article/supplementary material, further inquiries can be directed to the corresponding author.

## Ethics Statement

The studies involving human participants were reviewed and approved by Shahid Beheshti University of Medical Sciences. The patients/participants provided their written informed consent to participate in this study.

## Author contributions

HK, AH, MR, and AS conducted the research, analyzed the data, and performed statistical analysis. HS, M-AG, and MS contributed to the writing, design, and revision of the manuscript. AH contributed to the research design. All authors contributed to the article and approved the submitted version.

## Funding

This work was supported financially by the National Nutrition and Food Technology Research Institute, Shahid Beheshti University of Medical Sciences, Tehran, Iran (Grant Number: 1000328).

## Conflict of interest

The authors declare that the research was conducted in the absence of any commercial or financial relationships that could be construed as a potential conflict of interest.

## Publisher's note

All claims expressed in this article are solely those of the authors and do not necessarily represent those of their affiliated organizations, or those of the publisher, the editors and the reviewers. Any product that may be evaluated in this article, or claim that may be made by its manufacturer, is not guaranteed or endorsed by the publisher.
